# SLC7A5 Functions as a Downstream Target Modulated by CRKL in Metastasis Process of Gastric Cancer SGC-7901 Cells

**DOI:** 10.1371/journal.pone.0166147

**Published:** 2016-11-15

**Authors:** Junqing Wang, Xiaochun Fei, Weize Wu, Xuehua Chen, Liping Su, Zhenggang Zhu, Yunyun Zhou

**Affiliations:** 1 Department of Surgery, Ruijin Hospital, Shanghai Jiao Tong University School of Medicine, Shanghai, People’s Republic of China; 2 Shanghai Key Laboratory of Gastric Neoplasms, Ruijin Hospital, Shanghai Jiao Tong University School of Medicine, Shanghai, People’s Republic of China; 3 Shanghai Institute of Digestive Surgery, Ruijin Hospital, Shanghai Jiao Tong University School of Medicine, Shanghai, People’s Republic of China; 4 Department of Pathology, Ruijin Hospital, Shanghai Jiao Tong University School of Medicine, Shanghai, People’s Republic of China; 5 Department of Data Science, University of Mississippi Medical Center, Jackson, Mississippi, United States of America; Wayne State University, UNITED STATES

## Abstract

SLC7A5, who is also named LAT-1, has been validated as a promoter regulated by miRNA-126 in our previous research for gastric cancer cells. However, the mechanisms driving SLC7A5 to affect the bio-function of gastric cancer cells are unclear, remaining us lots of to elucidate. The aim of this study is to investigate the regulating effect of CRKL, one of the critical genes involving with gastric cancer progression, on SLC7A5 expression. By studying the gastric cancer cell lines and clinical pathological specimens, we found that the expression of SLC7A5 was significantly correlated to CRKL. By depleting CRKL in gastric cancer SGC-7901 cells, the SLC7A5 expression was impaired, and the invasion and migration of SGC-7901 cells were suppressed. Ectopic expression of SLC7A5 could drastically rescue the phenotypes induced by CRKL depletion in this study. Accordingly, we conclude that SLC7A5 functions as a promoter in gastric cancer metastasis, and CRKL could be one of its regulators modulating the expression of SLC7A5 and consequentially affect the metastatic feature of SGC-7901 cells. The findings in this study indicate a regulation relationship between CRKL and SLC7A5, and provide useful evidence for gastric cancer therapeutic strategies.

## Introduction

Gastric cancer (GC) is one of the most common digestive malignant tumors of human beings, especially in Asian people. The current 5-year survival rate of individuals with gastric cancer is ~24%, which reflects the aggressive behavior of this tumor [1, 2]. Metastasis is a major cause leads to high mortality rate and poor prognosis of GC patients. Median survival time of GC patients with local advanced invasion or metastasis is less than 12 months so far, and this leaves researchers and clinical doctors a great challenge[3, 4]. In the progress of research on tumorigensis, lots of genes have been discovered concerning with GC initial, process, invasion and metastasis. However, very few findings have been intensively studied for practical application clinically. Thus, investigations of the application in those functional genes benefit for therapeutic strategy of GC treatment.

As acknowledged, cancer cells take up large amounts of amino acids to maintain survival and to conduct aggressive malignant bio-behaviors. Solute carrier-7A5 (SLC7A5), who is also named L-Type amino acid transporter (LAT-1), is a member of system L-type transporters [5]. SLC7A5 gene locates at chromosome 16q24.3, and the protein product mediates large neutral amino acid transportation across cell membranes in a Na^+^ independent manner, which could supply essential amino for somatic cells [6, 7]. Accumulative evidences have indicated that SLC7A5 is essential for both normal and malignant cells to maintenance, and aberrant high expression of SLC7A5 has been observed in a variety of malignant cells than that in normal cells, including colon cancer, prostate cancer, pulmonary cancer and esophageal cancer [8–11].

Our previous research showed a clearly high expression of SLC7A5 in both gastric cancer tissues and cell lines. We found that high SLC7A5 expression is associated with GC clinicopathologic features such as tumor size, lymph node metastasis, local invasion and TMN stages. By knocking down SLC7A5 in gastric cancer SGC-7901 cells, the cell proliferation was impaired along with a significant arrest of G0/G1 phase of cell cycle. And motility of SGC-7901 cells was suppressed by observing through invasion and migration assay [12]. With intensive studies on SLC7A5, we further discovered that SLC7A5 is one of the targeted genes of microRNA-126 (miR-126), a pivotal post-transcriptional tumor suppressing microRNA in GC, by using dual-luciferase reporter assay. Thus, SLC7A5 could be a potential target for GC therapeutic treatment. However, we are interested to know if there exists other potential regulation upstream molecular driving for SLC7A5, which could provide us more information of the mechanism to affect GC cells’ motility.

CRKL, which is a V-crk avian sarcoma virus CT10 oncogene homolog-like adapted protein of CRK family, was confirmed by us previously as a promoter of GC[13]. Genes of this family encode adapter proteins mediating cell signaling transduction in a wide range of cell bio-function involving in physiological and pathological cell proliferation, survival, adhesion and migration. Dysfunction of CRKL plays key roles of a variety of human diseases including human malignancies, e.g. chronic myelogenous leukemia, colon cancer and prostate cancer [14–17]. However, we understand limited information of CRKL in GC process.

In the present study, we modulated the expression of CRKL in SGC-7901 cells, and observed that the depletion of CRKL in gastric cancer SGC-7901 cells was companied with a down-regulation of SLC7A5 at both mRNA stage and protein stage, which suggests SLC7A5 as a downstream gene of CRKL. By comparing the CRKL and SLC7A5 expression in real GC patients’ tissues from GSE13911 of GEO database, we also found a significantly positive correlation between them. We discovered that the suppression by depleting CRKL in SGC-7901 cells could be significantly rescued by over-expressing SLC7A5, especially in cell invasion and migration. Thus, we concluded that SLC7A5 could be a potential target for GC therapeutic treatment

## Materials and Methods

### Surgical specimens and cell culture

Seventy-two pairs of gastric cancer specimens and adjacent non-cancerous tissues were collected from GC patients who received radical gastrectomy without preoperative therapy at the Department of Surgery, Ruijin Hospital, Shanghai Jiao Tong University School of Medicine during 2011–2014. This study has been approved by the Institutional Review Boards of Ruijin Hospital, Shanghai Jiao Tong University School of Medicine, and the clinical specimens have been treated under approval of this ethics committee. The participants had provided the written consent to this study.

As we validated previously, both CRKL and SLC7A5 are highly expressed in gastric cancer SGC-7901 cells, we selectively chose SGC-7901 cells for study and the immortalized gastric epithelium cell line (GES-1) were used as control. Both cell lines were purchased from Shanghai Cell Bank (Shanghai, China), and were cultured in RPMI 1640 supplemented with 10% heat-inactivated fetal bovine serum (FBS), 100 ug/ml streptomycin and 100U/ml Penicillin in a humidified cell incubator at 37℃ with an atmosphere of 5% CO_2_.

### Confocal microscopy measurement, Western blot analysis and immunohistochemistry analysis

Antibodies against CRKL, SLC7A5 and GAPDH (Santa Cruz, USA), and horseradish peroxidase-conjugated secondary antibody (Abcam, USA) were purchased.

Cells were fixed with 4% paraformaldehyde and were incubated with antibodies according to the manufactory instruction (1:50). Cells above were examined under confocal microscopy. Collection of emission was from 488 nm to 561 nm.

RIPA buffer containing Protease Inhibitor Cocktail (Pierce, USA) was used to lyse cells. The protein concentration was measured by BCA protein Assay Kit (Pierce, USA). The proteins were electrophoresed and electrotransfered. Antibodies (1:1000) and GAPDH (1:5000) were probed, and further probe was carried out by using horseradish peroxidase-conjugated secondary antibody.

Tissues of formalin-fixed paraffin-embedded surgical specimens were constructed. Detection of CRKL and SLC7A5 was performed on paraffin sections (4μm) with the anti-SLC7A5 antibody (1:1000). The immune complex was visualized with the DakoREAL™ EnVision™ Detection System, Peroxidase/DAB, Rabbit/Mouse (Dako, Denmark), according to the manufacturer’s procedure. The nuclei were counterstained with hematoxylin. The sections were examined by two professional pathologists individually. GAPDH was used as loading control. RIPA buffer containing Protease Inhibitor Cocktail (Pierce, USA) was used to lyse the cells, and the protein concentration was measured by BCA Protein Assay Kit (Pierce, USA). Proteins were electrophoresed and electrotransfered. Antibodies (1:1000) and GAPDH (1:5000) were probed, and horseradish peroxidase conjugated secondary antibody was used for further probe. Protein quantity was detected by using GAPDH as loading control.

### RNA isolation and RT-PCR assay

Total RNA was extracted from cell lines by using regent (Invitrogen, USA) following the manufactory instructions. The first-strand cDNAs were synthesized through High-Capacity cDNA Reverse Transcription Kit (ABI, USA). RT-primers of MALAT1 and SF2/ASF were synthesized by Sangon Biotech Company (Shanghai, China) as follow: 1) CRKL: 5’-AGCAATCCAGAAAAGAGTACCC-3’ (forward) and 5’-TTCACTTCGCCTTCCCAC-3’ (reverse); 2) SLC7A5: 5’-CTATCACCTGGGCGTCATG-3’(forward) and 5’-TGGATCATGGAGAGGATGGAG-3’ (reverse). Real-time quantitative polymerase chain reaction (RT-PCR) was carried out according to TagMan Gene Expression Assays protocol (ABI, USA).

### Cell transfection

SGC-7901 cells were transfected with pGPU6/GFP/Neo vectors (GenePhrma, Shanghai, China) containing shRNA against CRKL mRNA or SLC7A5 mRNA by using Lipofectamine 2000 (Invitrogen, USA), and non-containing ones were used as negative control. Cell were cultured and selected in medium containing 400μg/ml G418 (Santa Cruz, USA), and were cultured and maintained in medium containing 200μg/ml G418.

Recombinant adenovirus Ad5/F35 (Ad5/F35-SLC7A5) was constructed for up-regulatingSLC7A5,and Ad5/F35-Null was used as negative control (GenePhrma, Shanghai, China). SGC-7901 cells which have been transfected with pGPU6/GFP/Neo/CRKL vectors were further transfected with Ad5/F35-SLC7A5 or Ad5/F35-Null to detect the rescuing effect of SLC7A5 in CRKL depleted cells.

Stable transfected cells above were validated by RT-PCR and confocal microscopy measurement compared with the negative control cells.

### Cell proliferation assay

The SGC-7901 cells transfected and the control ones were collected and were respectively cultured in 96-well microtiter plates in triplicate and incubated for 5 days at 37℃ with an atmosphere of 5% CO_2_. OD was measured by WST (Water-soluble trtrazolium salt USA) assay by using CCK8 Assay Kit (Dojindo, Japan) according to the protocol. The curves of cell proliferation were plotted.

### Cell migration and invasion assay

Cell migration or invasion assay was carried out by using the QCMTM 24-Well Colorimetric Cell Migration or Invasion Assay Kit (Millipore, USA). 3×10^4^ stable transfected SGC-7901 cells in 300μl serum free medium were added to the upper chamber, and 10% FBS-containing medium was used as chemoattractant in the lower chamber. ECMatrix^TM^ was pre-coated to the upper chamber for invasion assay. And cells on the bottom of the membrane were stained and checked after 24h for migration or 48h for invasion.

### Statistical analysis

Statistical analysis for 72 specimens in Table 1 was carried out by SPSS 18.0 and GraphPad Prism 5.0. P values were calculated by paired t-test and Fisher’s exact text. Those P values < 0.05 were considered statistically significant.

**Table 1:** The correlation between expression characteristic of CRKL and SLC7A5 in GC specimens and GC clinicopathologic features.

Clinicopathologic parameters	CRKL expression		P *	SLC7A5 expression		P *
	(n = 72)			(n = 72)		
	Low (n = 21)	High(n = 51)		Low (n = 25)	High(n = 47)	
**Age (years)**						
≤60	35(49%)	8(38%)	0.509	11(44%)	23(49%)	0.806
>60	37(51%)	13(62%)		14(56%)	24(51%)	
**Gender**						
Male	43(60%)	15(71%)	0.37	13(52%)	28(60%)	0.62
Female	29(40%)	6(29%)		12(48%)	19(40%)	
**Diameter (cm)**						
≤5	37(51%)	16(76%)	0.038	16(64%)	18(38%)	0.049
>5	35(49%)	5(24%)		9(36%)	29(62%)	
**Location**						
Distal third	45(63%)	11(52%)	0.76	14(56%)	30(64%)	0.614
Middle or proximal third	27(37%)	10(48%)		11(44%)	17(36%)	
**Histologic Classification**						
Poorly differerntial	34(47%)	9(43%)	0.792	9(36%)	19(40%)	0.654
Middle/well differerntial	18(25%)	7(33%)		11(44%)	15(32%)	
Signer ring cell cancer	11(15%)	3(12%)		3(12%)	8(17%)	
Mucinous asenocainoma	9(13%)	3(12%)		2(8%)	7(11%)	
**Location invasion**						
T1, T2	28(39%)	9(43%)	0.002	16(64%)	15(32%)	0.009
T3, T4	44(61%)	12(57%)		9(36%)	32(68%)	
**Lymph node metastasis**						
No	24(33%)	10(48%)	0.004	15(60%)	12(26%)	0.016
Yes	48(67%)	11(52%)		10(40%)	35(74%)	
**TNM stage**						
I, II	37 (51%)	11(52%)	0.001	15(60%)	14(30%)	0.045
III, IV	35 (49%)	10(48%)		10(40%)	33(70%)	

CRKL and SLC7A5 expression level associated with clinicopathologic features in GC patients,including age, gender, tumor size, tumor location, histologic classification, local invasion, lymph node metastasis and TNM stage. Statistically significance was assessed by Fish’s exact text.

*:Significant difference with *P* value<0.05.

The whole genome mRNA microarray expression of from GSE13911 with 38 tumor samples and 31 non-tumor samples were downloaded from NCBI GEO database. We applied the quantile normalization to adjust the global noise among different samples. Then, we screened the most significantly differential expressed genes (*p-*value <0.05) between tumor and normal samples by R “limma” package. Within the differential expressed genes, the top correlated genes with CRKL was selected by Pearson Correlation test with *p-*value < 0.05 and 2 fold change between tumor and normal. The heatmap was generated by “ggplot2” package in R. SLC7A5 is one of the correlated genes of CRKL based on Pearson correlation test.

For the survival analysis of CRKL and SLC7A5, we use online tool (http://www.kmplot.com/gastric) to generate Kaplan–Meier curves, which include 876 gastric cancer patients with available clinical data. For the expression of the genes, each percentile of expression between the lower and upper quartiles was computed and the best performing threshold was used as the final cutoff for the Univariate Cox regression analysis. Kaplan–Meier survival plot were downloaded from their website and the hazard ratio with 95% confidence and P Value were calculated.

## Result

### Expression of CRKL and SLC7A5 are up-regulated simultaneously in SGC-7901 cells

**Fig 1:**
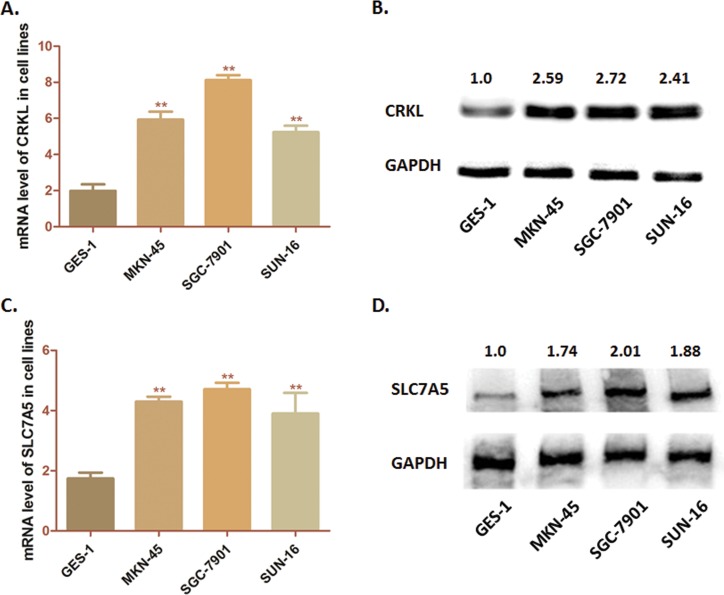
Expression of CRKL and SLC7A5 in cell lines. (A) Analysis of transcription level of CRKL in cell lines by RT-PCR. The mRNA levels of CRKL in three cell lines (MKN-45, SGC-7901 and SUN-16) was significantly higher than that in GES-1 cells (**P<0.01) (B) Detection of protein expression of CRKL in cell lines by western-blot analysis. CRKL was significantly over-expressed in GC cells compared with GES-1 cells. (C) RT-PCR was conducted to measure the mRNA levels of SLC7A5 in these three cell lines. SLC7A5 mRNA level was significantly higher than that in GES-1 cells (**P<0.01) (D) Western-blot analysis was carried out to measure the protein expression of CRKL. CRKL was significantly over-expressed in GC cells compared with GES-1 cells. The numbers above the blot indicate the normalized protein amounts relative to the negative control, as determined by densitometry. As histograms above shown, SGC-7901 cells presented a highest expression of both CRKL and SLC7A5 among the GC cell lines.

First, we detected the expression of CRKL and SLC7A5 in 3 GC cell lines (MKN-45, SGC-7901 and SUN-16) and one immortalized gastric epithelium cell line (GES-1) through RT-PCR and Western blot analysis. As Fig 1A showed, both CRKL and SLC7A5 presented higher mRNA level in tumor cells than that of GES-1 cells. Simultaneously, the protein level of CRKL and SLC7A5 was significantly higher in GC cells than in GES-1 cells (Fig 1B). According to these results, we observed that CRKL and SLC7A5 share the similar expression characteristic in these three GC cell lines.

### SLC7A5 is highly expressed in GC tissues positively correlated with CRKL

**Fig 2:**
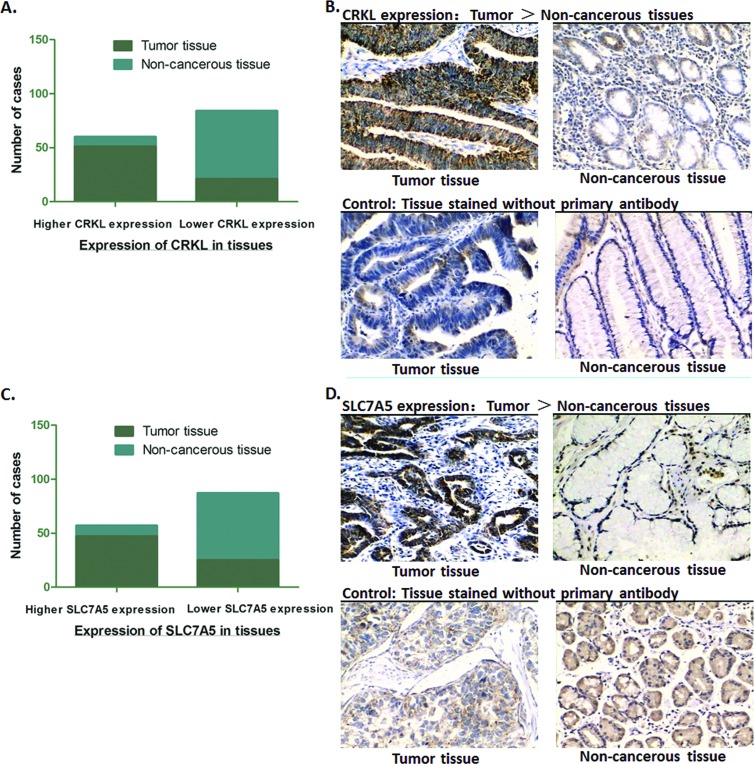
Expression of CRKL and SLC7A5 in GC tissues. (A) Immunohistochemical analysis showed that high expression of CRKL in 70.8% (51/72) of the tumor tissues, and low expression in 87.5% (63/72) the adjacent non-cancerous tissues. The expression of CRKL in GC tumor tissues was significantly higher than the adjacent non-cancerous tissues (**P<0.01). (B) Representative graph immunohistochemistry analysis (400×) for CRKL. Specimens stained without primary antibody was used for control. (C) Immunohistochemical analysis showed that high expression of SLC7A5 in 65.3% (47/72) tumor tissues, and low expression in 86.2% (62/72) the adjacent non-cancerous tissues. The expression of SLC7A5 in GC tumor tissues was significantly higher than the adjacent non-cancerous tissues (**P<0.01). (D) Representative graph immunohistochemistry analysis (400×) for SLC7A5. Specimens stained without primary antibody was used for control.

Considering what we observed in GC cells lines, we wonder if there exists the same trend in real patients’ tumor tissues. Analysis of the expression of both CRKL and SLC7A5 in 72 tumor specimens by IHC was conducted, compared with the adjacent non-cancerous tissues. According to the content of CRKL, the paired specimens were divided into two groups: CRKL low expression group and CRKL high expression group. In tumor specimens, 70.8% (51/72) of the cases showed ‘high expression’ of CRKL, and in non-cancerous tissues, only 12.5% (9/72) of the cases showed high CRKL expression, which was consistent with our previous finding that CRKL frequently expresses higher in GC tumor tissues. As for SLC7A5, similar” with CRKL, specimens were divided into SLC7A5 high expression group and SLC7A5 low expression group. We observed that 65.3% (47/72) of the tumor specimens showed obviously high expression of SLC7A5, while, in the non-cancerous tissues, only 13.8% (10/72) cases showed high SLC7A5 level. Thus, we suggest that both CRKL and SLC75A are expressed significantly higher than that of adjacent non-cancerous tissues (*P*<0.01) (Fig 2).

**Fig 3:**
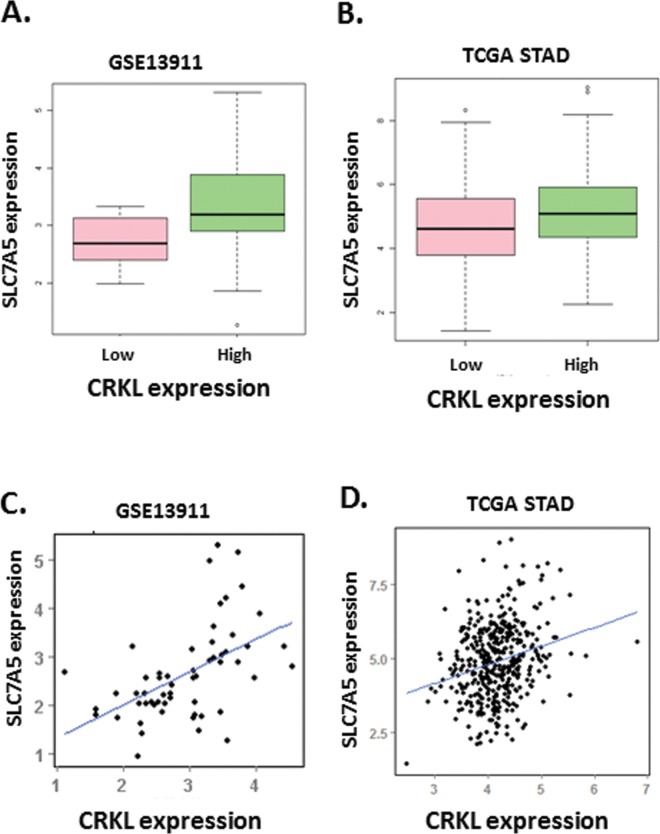
The results of data mining shows positive correlation between CRKL and SLC7A5 and illustrates a high risk of mortality for GC patients with high expression of ABCG2 and CRKL. (A~C) Dataset GSE13911 was downloaded from NCBI GEO database. (A) The heat map presents a frequently highly expression of CRKL and SLC7A5 mRNA in GC patients specimens. (B) The plots demonstrate markedly higher expression of CRKL in tumor tissues than that of non-cancerous tissues. (C) The Plots shows a significant positive correlation between CRKL and SLC7A5 (*P*<0.01). (D) Patients with high correlated expression of CRKL in tumor tissue showed a significant tendency towards poor prognosis and high mortality (*P*<0.01). (E) Patients with high correlated expression of SLC7A5 in tumor tissue showed a significant tendency towards poor prognosis and high mortality (*P*<0.01).

According to the observation, we are interested to know whether the expression characteristic of CRKL and SLC7A5 was also correlated in other independent mRNA expression dataset of GC. As Fig 3A and 3B shown, CRKL presented a significantly higher expression (adjusted p-value 1.24e-07) in tumor samples compared with normal calculated from “limma” package. And mRNA microarray data analysis indicated that the expression of SLC7A5 was significantly positive correlated with CRKL according to the *p-*value as 4.467e-05 (Fig 3C). Results above, combining with what we observed in tumor cells and tissues, strongly illustrate a positive correlation between CRKL and SLC7A5 in gastric cancer cells.

### The expression of SLC7A5 correlated with CRKL involves with poor prognosis and clinicopathologic features

As both CRKL and SLC7A5 presented high expression in GC, we analyzed the GC clinicopathologic features of the 72 cases, compared with the expression of these two genes. As shown in Table 1, no correlation was observed between neither CRKL nor SLC7A5 and the patients’ age, gender, tumor location (*P*>0.05). While, patients with either CRKL or SLC7A5 over-expressed in tumor tissues exactly presented a tendency of large tumor size (*P*<0.05), deeper local invasion (*P*<0.05), much more frequent lymph node metastasis (*P*<0.05) and more advanced TNM stages (*P*<0.05), which indicated poor prognosis compared with the patients with relatively lower CRKL and SLC7A5 expression.

Additionally, we conducted survival analysis of CRKL and SLC7A5 by applying online Kaplan–Meier plotter tools. Patients with high correlated expression of either CRKL or SLC7A5 in tumor tissue showed a significant tendency towards poor prognosis and high mortality (*P*<0.05). These results also independently confirmed that SLC7A5 and CRKL are associated with poor prognosis (Fig 3C–3E).

### Depletion of CRKL induces decreasing of SLC7A5 and suppresses cell proliferation and motility of SGC-7901 cells

**Fig 4:**
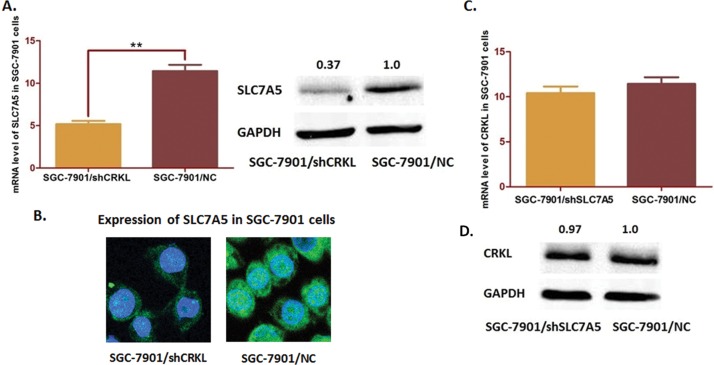
Depletion of CRKL in SLC7A5 cells transfected by pGU6/Neo/siCRKL vector. (A) Immunochemistry staining of cells was carried out to exam the stable transfected SGC-7901 cells (400×). The staining intensity of SGC-7901 cells transfected with CRKL-shRNA (SGC-7901/shCRKL) were significantly decreased compared with the negative control cells. (B) The QRT-PCR assay results indicated that CRKL mRNA level was significantly decreased compared with the negative control (**P<0.01). (C) The Western blot analysis results presented a significant suppression of CRKL expression after transfection (*P<0.05).

**Fig 5:**
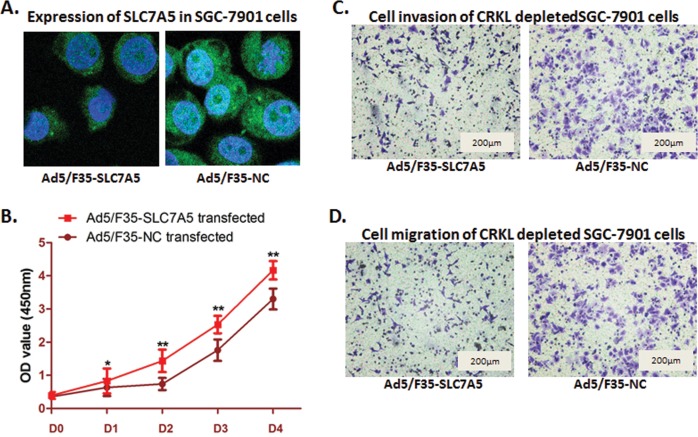
Effects of CRKL depletion on SLC7A5 expression in SGC-7901 cells. (A) RT-PCR and Western-blot analysis of the SGC-7901 cells. Both mRNA and protein levels of SLC7A5 in SGC-7901 cells were significantly decreased when CRKL was depleted (**P<0.05). (B) Confocal microscopy measurement shows that the expression of SLC7A5 in CRKL depleted SGC-7901 cells. (C) Down-regulating SLC7A5 in SGC-7901 cells induces no significant change of CRKL at neither mRNA nor protein level (*P*>0.05).

Regarding that CRKL and SLC7A5 are correlated expressed in GC, we further investigated the potential regulating relationship between them. PGPU6/GFP/Neo vectors were transfected into the SGC-7901 cells and to deplete CRKL (Fig 4A–4C). Interestingly, SLC7A5 expression was significantly decreased when CRKL was depleted at both mRNA stage and protein stage (Fig 5A). In contrast, SLC7A5 depletion by transfection in SGC-7901 cells seemed no significant change in CRKL expression (Fig 5B and 5C). These observations indicated that CRKL should affect SLC7A5 as an up-stream molecule through some transcriptional mechanism.

**Fig 6:**
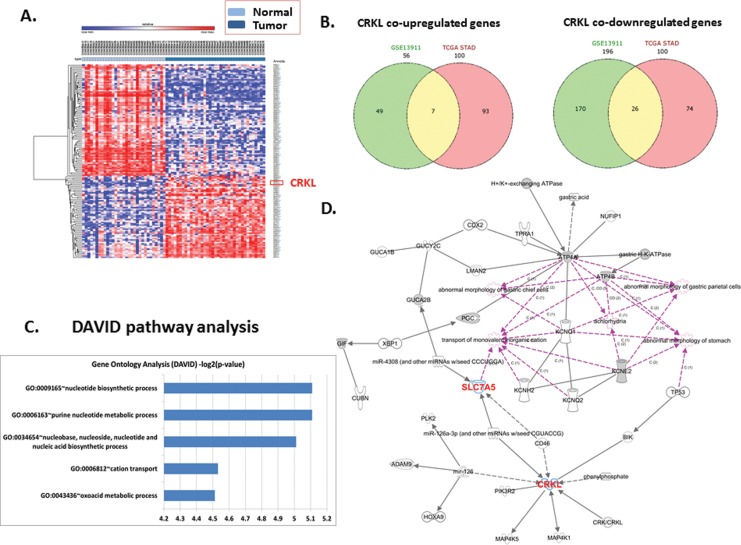
Effects on cell proliferation and cell motility by depleting CRKL in SGC-7901 cells. (A) The cell proliferation was determined by WST assay when CRKL was depleted in SGC-7901 cells. The ability of cell proliferation of SGC-7901 cells was significantly decreased. The results are means of three independent experiments SD. (**P<0.01). (B) Trans-well assay was carried out to detect the effect of CRKL depletion on cell invasion and migration of SGC-7901 cells. Depleting CRKL induced a significantly decreased number of cells invaded through the low chamber (265±17 cells per field for the control group, 143±13 cells per field for CRKL depleted SGC-7901 cells, **P<0.01). (D). Depleting CRKL induced a significant decreased number of cells migrated into the ECMatrixTM precoated (263±15 cells per field for the control group, 148±18 cells per field for CRKL depleted SGC-7901 cells, **P<0.01).

As cell functional assays indicated, depleting CRKL in SGC-7901 cells significantly inhibited the cell proliferation (Fig 6A). The motility of SGC-7901 cells, was markedly suppressed when down-regulating CRKL through trans-well assay (Fig 6B and 6C). For invasion assay, CRKL depletion led to a significantly decreased number of cell invading into the lower chamber (265±17 cells per field for the control group, 143±13 cells per field for CRKL depleted SGC-7901 cells, *P*<0.01). And the migration assay also indicated an obvious decline of SGC-7901 cell number transited into the lower chamber after CRKL depletion (263±15 cells per field for the control group, 148±18 cells per field for CRKL depleted SGC-7901 cells, *P*<0.01). Results of cell functional assay strongly illustrated significant depression effects on both GC cells proliferation and cell motility, which suggested CRKL as a remarkable target hopefully for GC treatment. However, how CRKL functions in GC cells and what is the downstream regulating mechanism remain unclear.

### Ectopic expression of SLC7A5 rescues the phenotype induced by CRKL depleting

**Fig 7:**
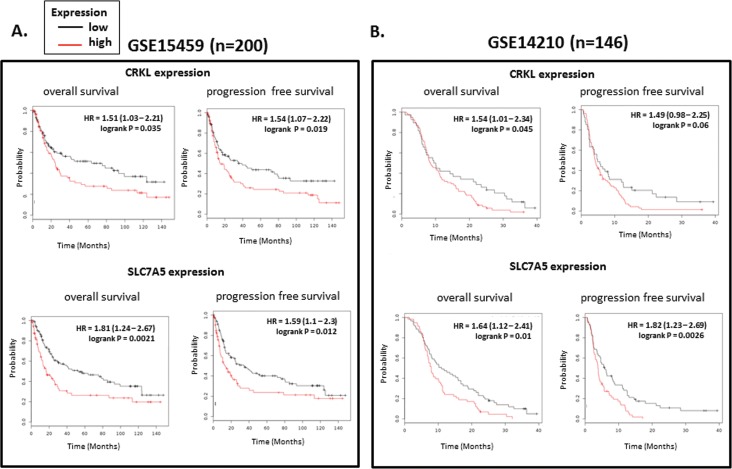
Ectopic expression of SLC7A5 reversed the biological phenomena induced by CRKL depletion in MKN-45 cells. (A) Recombinant adenovirus Ad5/F35 was transfected to up-regulate the expression of SLC7A5 in CRKL depleted SGC-7901 cell. Confocal microscopy measurement showed that SLC7A5 was up-regulated after transfection. (B) WST assay was carried out to determine the cell proliferation ability after SLC7A5 ectopicly expressied in SGC-7901 cells. Cell proliferation suppression induced by CRKL depletion in was significantly reversed by introducing SLC7A5. The results are means of three independent experiments ±SD. (**P<0.01,*P<0.05). (B) Invasion assay showed a significant increase of cells invaded into the lower chamber (135±11 cells per field for the control group, 228±19cells per field for vector transfected SGC-7901 cells, **P<0.01). (C) Migration assay presented a significant increase of SGC-7901 cells migrated from the upper chamber to the lower one (152±13cells per field for the control group, 237±14 cells per field for vector transfected SGC-7901 cells, **P<0.01).

As SLC7A5 was correlated with CRKL, and the expression of SLC7A5 was decreased when CRKL depleted, we hypothesized that SLC7A5 could be an effective downstream genes regulated by CRKL in GC cells. We constructed recombinant adenovirus Ad5/F35-SLC7A5 to up-regulate SLC7A5, whose expression had been suppressed when CRKL was depleted in SGC-7901 cells. Cell functional assay was carried out after SLC7A5 up-regulation was validated by confocal microscopy measurement (Fig 7A). As Fig 7B–7D shown, cell proliferation was significantly rescued when SLC7A5 up-regulated (*P*<0.05), and the SGC-7901 cells counted transferred from the upper chambers into the lower chambers in both invasion assay and migration assay was obviously increased(For invasion assay: 135±11 cells per field for the control group, 228±19 cells per field for vector transfected SGC-7901 cells, *P*<0.01; For migration assay: 152±13cells per field for the control group, 237±14 cells per field for vector transfected SGC-7901 cells, *P*<0.01). These results indicated that up-regulating SLC7A5 could rescue the phenotype in SGC-7901 cells that induced by depleting CRKL. Therefore, SLC7A5 could be one of the downstream genes of CRKL in SGC-7901 cells, which leads the tumor cells to proliferate, invade and migrate.

## Discussion

Progress on laboratory studies reveals the biological behaviors and correlated mechanisms of gastric cancer these recent decades. Variety of molecules involve in GC tumorigensis, process, which have been discovered related with different cellular mechanisms, could be mighty targets in GC diagnosis and treatment. In this study, we suggested two molecules, CRKL and SLC7A5, which have positive correlation in both GC tissues and cell lines, as potential targets in GC therapeutic treatment. And we illustrated some of the mechanism that CRKL and SLC7A5 drive to promote GC cells proliferation, invasion and migration.

Accumulating evidence has been reported and suggested that dysfunction of CRKL as a key factor in variety of human diseases, including inflammation and carcinomas [18–20]. In our previous study, we also validated CRKL as a promoter in GC, induces GC cells proliferation and motility. And the mechanism of how CRKL affects GC cells to be more aggressive is one of the interesting subjects to illustrate for our further discussion about the possibility regarding CRKL as a therapeutic target. SLC7A5 is a member of L-type transporters, which characterizes high affinity for nutritionally essential amino acid, and transports the components from extra-cellular environment to intra-cellular structure through a sodium-independent method [21, 22]. SLC7A5 is essential for cell functions in numbers of carcinomas [23], and has been detected highly expressed in prostate cancer, ureteral cancer and colorectal cancer [24–26]. We has validated that SLC7A5 was over-expressed in GC, and suggested SLC7A5 as a potential oncogene involved with GC process. However, there has no description and report yet about the relationship between CRKL and SLC7A5 before. And we wonder if we could took an insight on these two functional genes in GC, which could provide us more evidence to treat GC by targeting CRKL and SLC7A5

In this study, firstly, we found that both CRKL and SLC7A5 were over-expressed in GC cell lines. CRKL and SLC7A5 are correlated in both mRNA stage and protein stage. Secondly, the expression status in cells is supposed to be validated in real tumor tissues. We detected 72 pairs of tumor specimens with the adjacent non-cancerous tissues. Similarly, most of the tumor tissues expressed in high level for both CRKL and SLC7A5, which is consistent with the result discovered in GC cell lines. This information provides us confident evidence that CRKL and SLC7A5 might have some functional relationship in GC process worthy to be illustrated.

We further validated our hypothesis by studying the database downloaded from NCBI GEO dataset to confirm the relationship between CRKL and SLC7A5 in published GC patients’ dataset. By analyzing the mRNA expression of 38 gastric tumor and 31 adjacent normal samples from GSE13911, we found that CRKL is one of the top ranked differential genes that over expressed in tumor specimens compared with normal tissue. Meanwhile, the expression of CRKL and SLC7A5 were significantly positively correlated in patients’ samples, which strongly supported the observation we found in that of 72 paired specimens and three cell lines. We suggested the expression of CRKL and SLC7A5 may functional related in GC.

However, we are interested to know the detailed regulating relationship between CRKL and SLC7A5. Depletion of CRKL was carried out in SGC-7901 cells, which presented the highest level of both CRKL and SLC7A5 among the three cell lines. Markedly, cell proliferation of SGC-7901 cells was suppressed when CRKL was knocked down, and the number of cells transferred into the lower chambers in trans-well assay, which represented the ability of cell motility, was significantly decreased. These results indicated a strong promotional effect of CRKL on GC cell proliferation, invasion and migration. Interestingly, we discovered an observable decrease of SLC7A5 at mRNA and protein stages when CRKL was depleted. And we also down regulated SLC7A5 expression by using shRNAs in SGC-7901 cells. There was no significant alteration observed in CRKL expression when SLC7A5 was down regulated. Thus, we suggest that SLC7A5 could be a downstream genes regulated by CRKL in GC process.

In addition, recombinant adenovirus Ad5/F35 was transfected into the SGC-7901 cells with CRKL depletion. The CCK8 assay showed a significant increase of cell proliferation which suppressed by CRKL depletion. And the invaded and migrated cell numbers in the trans-well assay were raised compared with that of in CRKL depleted SGC-7901 cells. These results means the phenotypes that induced by CRKL depletion could be rescued through up-regulating SLC7A5, and further supported that SLC7A5 functions as a downstream gene of CRKL, which promotes GC cell proliferation and motility. Throughout what we found, it is clear that both CRKL and SLC7A5 are involved with the aggressive behavior of GC cells, including cell growth and motility. And these two genes could be potential targets, through which we could practice much more study to discuss the possibility to apply in GC targeted treatment.

Necessarily, further study should be conducted to discuss the mechanism CRKL regulating SLC7A5 in GC. We speculate that CRKL influences SLC7A5 through some kind of signaling pathway associated with transcriptional or post-transcriptional modulation according to the mRNA decrease of SLC7A5 when CRKL was depleted. Noticeably, several transcription factors have been mentioned potentially combining with the enhancer or promoter of SLC7A5 recently, such as MyC, GATA4 and HIF2a [27–29]. However, evidence involving in CRKL, SLC7A5 and related transcription factors is not sufficient. Herein, luciferase reporter assay and immunoprecipitation assay are required for detect whether CRKL could affect the transcriptional activity of SLC7A5 with an exact combination between the candidate transcription factors and SLC7A5 DNA sequence.

## Conclusion

In summary, in this study, both CRKL and SLC7A5 were over-expressed in gastric cancer cell lines and tissues. The expression of CRKL and SLC7A5 was strongly positively correlated, and the high level of CRKL and SLC7A5 suggested poor prognosis of GC patient combined with the clinicopathologic features. SLC7A5 is a downstream target of CRKL, by which the expression of SLC7AC could be altered. And modification of SLC7A5 level in GC cells could reversely affect the phenotypes induced by depleting CRKL. Thus, SLC7A5 functions as a promoter in gastric cancer metastasis, and CRKL could be one of its regulators modulating its expression and consequentially affects the metastatic feature of SGC-7901 cells. The findings here indicate a regulating relationship between CRKL and SLC7A5, and provide further evidence to advance new targets for gastric cancer therapeutic treatment.

## Supporting Information

S1 Fig Down-regulation of CRKL in SGC-7901 cells. (A) Immunochemistry staining demonstrates a significant decrease of CRKL expression in SGC-7901 cells by transfecting shRNA-CRKL. (B) QRT-PCR assay indicates a significant decrease of CRKL expression in SGC-7901 cells after transfecting shRNA-CRKL (**P<0.01). (C) Western-blot analysis indicates a significant decrease of CRKL expression in SGC-7901 cells after transfecting shRNA-CRKL.
(TIF)

S2 Fig Down-regulation of CRKL in SGC-7901 cells affects the cell proliferation and motility. (A) CCK8 assay shows that the cell proliferation was significantly inhibited through down-regulating CRKL in SGC-7901 cells. (B) Transwell assay indicates a significant inhibition of cell migration ability through CRKL down-regulation. (C) Transwell assay indicates a significant inhibition of cell migration ability through CRKL down-regulation.
(TIF)

## Abbreviations

GC: Gastric cancer
SLC7A5: Solute carrier-7A5
LAT-1: L-Type amino acid transporter
CRKL: V-crk avian sarcoma virus CT10 oncogene homolog-like adapted protein of CRK family
