# Supplementary figures and images for: SLC7A5 Functions as a Downstream Target Modulated by CRKL in Metastasis Process of Gastric Cancer SGC-7901 Cells
Source: PLoS One. 2016 Nov 15;11(11):e0166147. doi: 10.1371/journal.pone.0166147 (PMC5112787; doi:10.1371/journal.pone.0166147)

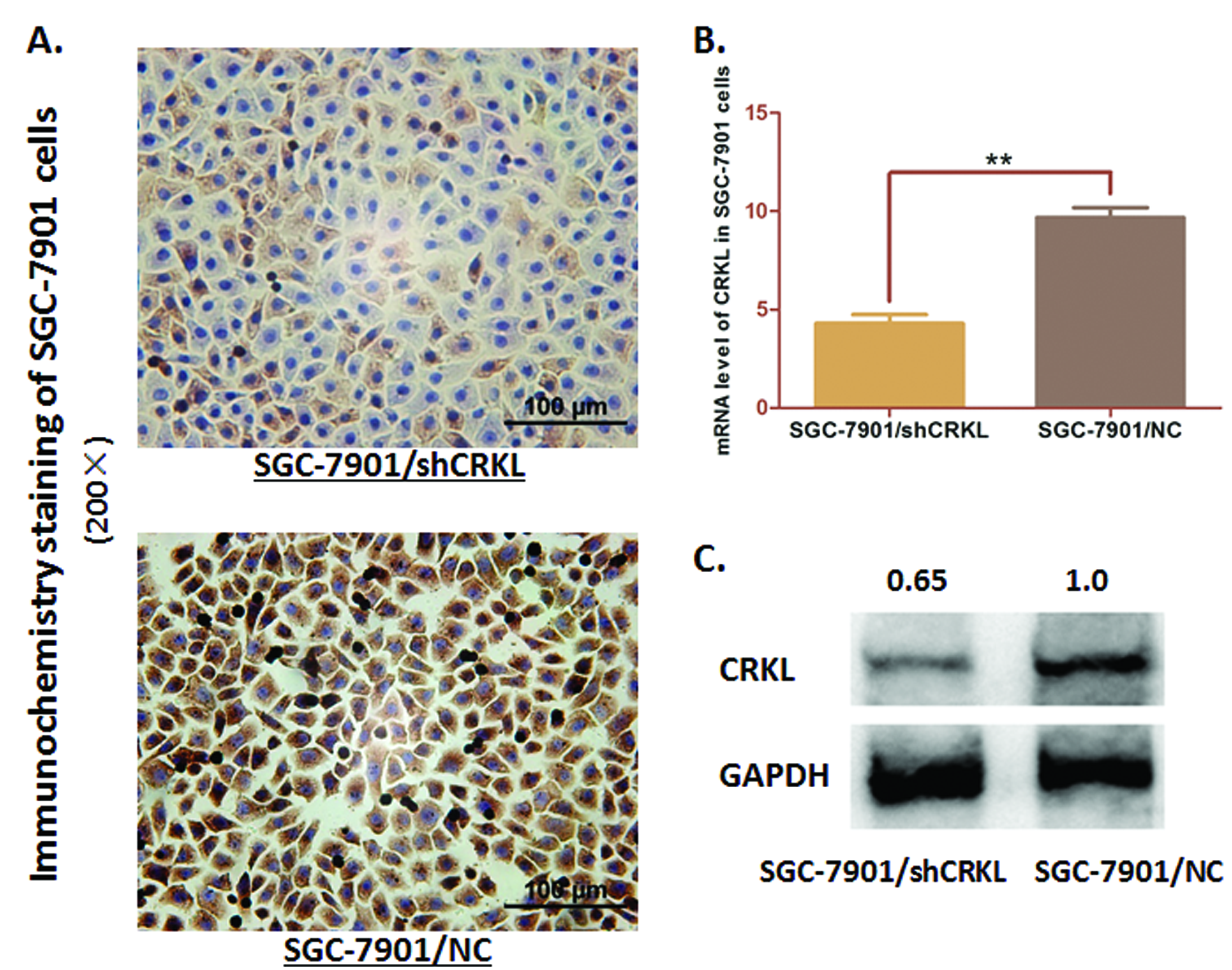

Supplement: S1 Fig — (A) Immunochemistry staining demonstrates a significant decrease of CRKL expression in SGC-7901 cells by transfecting shRNA-CRKL. (B) QRT-PCR assay indicates a significant decrease of CRKL expression in SGC-7901 cells after transfecting shRNA-CRKL (**P<0.01). (C) Western-blot analysis indicates a significant decrease of CRKL expression in SGC-7901 cells after transfecting shRNA-CRKL. (TIF) [file pone.0166147.s001.tif]

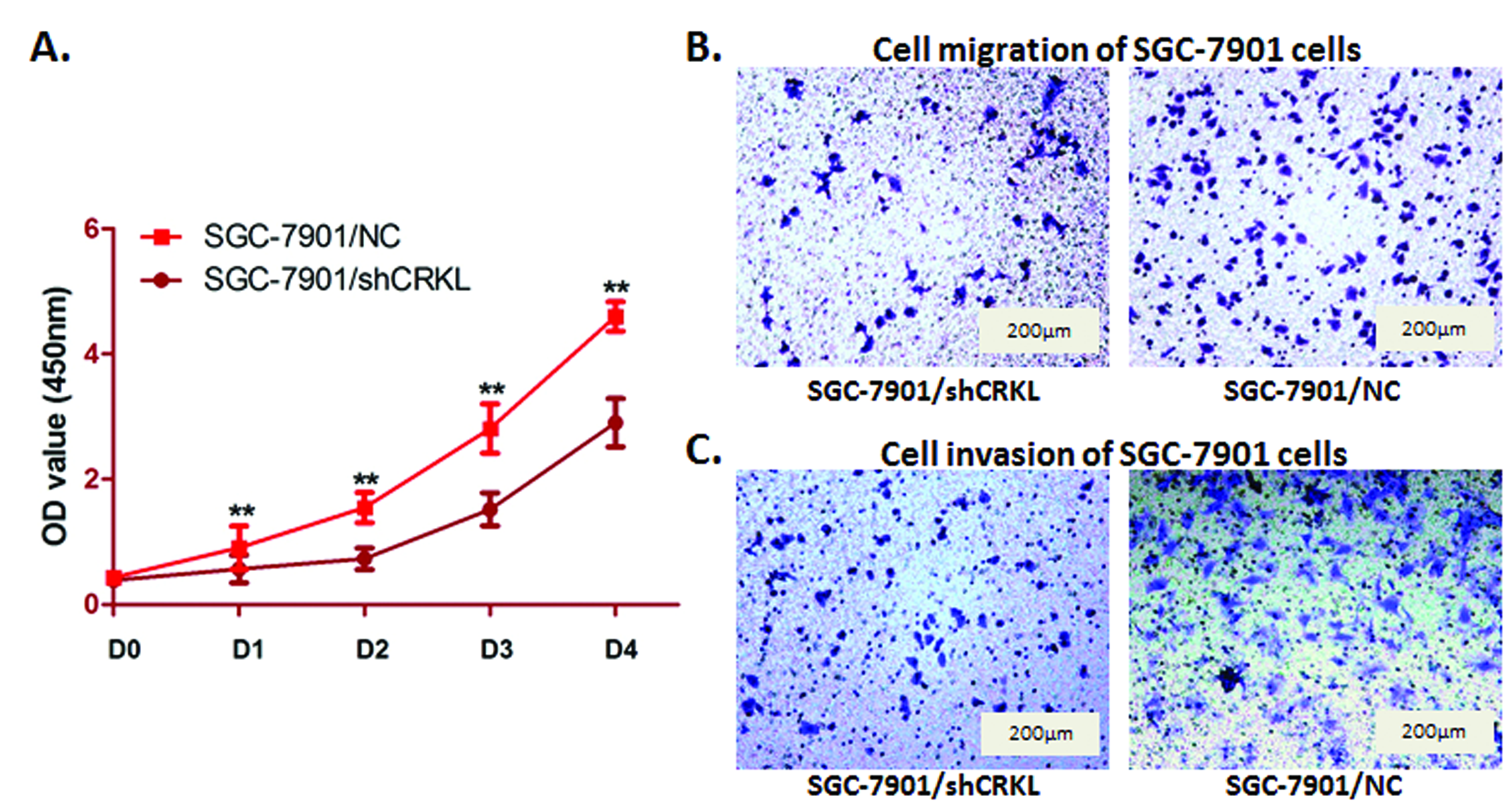

Supplement: S2 Fig — (A) CCK8 assay shows that the cell proliferation was significantly inhibited through down-regulating CRKL in SGC-7901 cells. (B) Transwell assay indicates a significant inhibition of cell migration ability through CRKL down-regulation. (C) Transwell assay indicates a significant inhibition of cell migration ability through CRKL down-regulation. (TIF) [file pone.0166147.s002.tif]
